# Barriers and opportunities to smallholder goat enterprise in Botswana

**DOI:** 10.1007/s12571-026-01648-7

**Published:** 2026-03-05

**Authors:** Lovemore C. Gwiriri, Honest Machekano, Andrew S. Cooke, Casper Nyamukondiwa, Andrews Safalaoh, Javier Ventura-Cordero, Paul Airs, Jan van Wyk, Patson Nalivata, Winchester Mvula, Virgil Joseph, Jonathan H. I. Tinsley, Michael R. F. Lee, Eric R. Morgan, Taro Takahashi

**Affiliations:** 1 Centre for Agroecology, Water and Resilience, Coventry University, Coventry, UK; 2 Net-Zero and Resilient Agriculture, Rothamsted Research, Okehampton, UK; 3 Botswana International University of Science and Technology, P. Bag 16, Palapye, Botswana; 4 Department of Veterinary Tropical Diseases, University of Pretoria, Private Bag X20, Hatfield, 0028 South Africa; 5 School of Natural Sciences, College of Health and Science, University of Lincoln, Lincoln, UK; 6 Department of Zoology and Entomology, Rhodes University, Makhanda, 6140 South Africa; 7 Centre for Environmental Policy, Imperial College of London, London, UK; 8 Lilongwe University of Agriculture and Natural Resources, Lilongwe, Malawi; 9 Institute for Global Food Security, School of Biological Sciences, Queen’s University Belfast, Belfast, UK; 10 School of Sustainable Food and Farming, Harper Adams University, Newport, Shropshire UK; 11 Agri-Food & Bioscience Institute, Hillsborough, UK; 12 Bristol Veterinary School, University of Bristol, Langford, UK

**Keywords:** Livestock, Agriculture, Ruminants, Gender, Veterinary

## Abstract

Smallholder households across rural sub-Saharan Africa often rely on goats to support their livelihoods, food security, and financial security, and as a risk mitigation strategy. Across the region, goat health and performance outcomes vary greatly, and it is often difficult to quantify the impact of the animal health-nutrition nexus on livelihood outcomes. Two socio-economic surveys were conducted to this end in Botswana. Firstly, a general cross-sectional survey of 787 rural households to assess the correlation between food and/or financial security and livestock practices. Secondly, a more nuanced survey of a sub-group of 44 goat-owning smallholders to understand the systemic interlinkages between livelihood outcomes, veterinary care, and goat management. While the importance of livestock to rural communities was confirmed by a positive association with improved food, financial and asset security, household variation was explained more by herd size and self-reported importance of goat ownership to households, than to goat health condition. Four main barriers were identified which negatively influenced goat ownership and herd survivability patterns: (1) low turnover rates and poor access to capital investment; (2) Limited use of supplementary feeds; (3) Poor access to veterinary services; and (4) Age and gender. Strategies to improve the productivity and sustainability of goat enterprises within the region should focus on overcoming these barriers, with the potential in doing so to improve food and financial security, and equality outcomes.

**Supplementary Information:**

The online version contains supplementary material available at 10.1007/s12571-026-01648-7.

## Introduction

The majority of smallholder farmers in sub-Saharan Africa (SSA) derive livelihoods from arid, peripheral regions on small pieces of land with informal tenure arrangements (Deininger et al., 2017). In such regions, agricultural systems are often labour-intensive due to limited access to capital and technology and, in turn, associated with poor yields and productivity (Williams et al., 2018). In addition, weather and climate change impose further pressures on food security and livelihood resilience (Cooke et al., 2024a; Perez et al., 2015).

Sustainable and resilient livelihoods are premised on establishing combinations of assets and activities that confer capacity for goat-based livelihoods to buffer (absorb change), self-organise (adapt or cope) and learn (transform) in the face of emerging shocks to maintain and optimise functional productivity (Obrist et al., 2010; Speranza et al., 2014; Perez et al., 2015). Thus, livelihood resilience in goat owning households is structured around the buffer capacity of goat production systems, how households are networked/organised to enable access to assets that optimise goat production, and the decisions to employ the assets to respond to emerging risks and shocks (learning capacity). Hence, to unpack the contribution of goats to sustainable and resilient livelihoods, we operationalise components of the Sustainable Livelihoods Framework (SLF) (Chambers & Conwary, 1992), which emphasizes the importance of the strength and diversity of assets in sustaining livelihoods. Here, how households leverage on, and access, available livelihood assets or capital (i.e. natural, human, financial, social, and physical) frames how smallholder goat holdings augment the broader livelihood strategies they pursue (DFID, 1999). Smallholder livestock systems have a cyclical relationship with these forms of assets, both relying on them and deploying strategic asset combinations to strengthen livelihood options. Goat systems are reliant on natural capital, particularly water, forage, and browse, to support the role of goats as tradeable asset for income diversification and non-tangible livestock benefits (Kaumbata et al., 2020; Kumar et al., 2010), thus contributing to integrated crop-livestock reliant livelihood strategies. In turn, well-managed livestock enterprises can support natural capital by promoting sustainable grazing practices that help maintain vegetation and soil health. Human capital, including knowledge, skills, and labour for husbandry and health management, is critical for sustaining productive systems and maintaining goat herds as forms of income insurance. Cross-breeding in goats to improve productivity and crop-livestock integration enables smallholder farmers to craft livelihoods on smaller pieces of land. Livestock enterprises contribute to building human capital not only through skills sharing and experiential learning, but also indirectly by strengthening financial capital, which can be reinvested in improved husbandry practices and cropping, as well as a coping strategy to supplement financial requirements for household healthcare and education. Many smallholder goat systems depend on communal grazing lands and cooperatives, benefitting from social capital while simultaneously buffering households from shocks such as drought-induced crop failures. Cooperation around livestock husbandry fosters trust and supports local marketing arrangements within communities, which can extend to other livelihood activities, communal resource management and crop-livestock marketing (Salmon et al., 2018). Similarly, physical capital such as kraals or bomas, are sources of manure that supports cropping, improves operational efficiency and income diversification. Thus, goats optimise conversion of physical capital to other assets, which helps diversify financial resources, reduce losses and minimise livelihood risks.

The capital stored in and built from livestock production plays a key livelihood buffer role for the ~ 52.5 million smallholder livestock farmers in SSA, by helping to buffer smallholders from shocks, such as adverse weather or economic difficulty, acting as a form of risk mitigation or insurance (Airs et al., 2023; Pica-Ciamarra et al., 2011). This is especially true in arid areas where: crop productivity is low, there is exposure to climatic extremes, soil quality is poor, and land availability is low (Joy et al., 2020; Kumar et al., 2010; Morales-Jerrett et al., 2020; Wodajo et al., 2020). Goats are popular in this regard, due to their hardiness, disease and thermal tolerance, dietary plasticity and subsequent low input costs (Mayberry et al., 2018; Medeiros et al., 2015; Zvinorova et al., 2016). Consequently, goats contribute to the socio-economic well-being and food security of resource-constrained households across SSA. Here, we explore empirical evidence for this by linking access to food with goat ownership.

Goats also play important gender and socio-cultural roles. Despite cultural differences throughout the region, women and youths typically assume primary roles in the ownership and husbandry of goats. This has recently increased as most governments in SSA are leveraging on goat ownership enterprise as both a poverty eradication and an employment opportunity tool for vulnerable groups in rural and peri-urban communities (Kaumbata et al., 2020; Khowa et al., 2023; Monau et al., 2020; Otieno, 2025). Therefore, this can provide a gateway to economic activity, whilst also supporting independence and food security (Bettencourt et al., 2015; Njuki & Sanginga, 2013; Wodajo et al., 2020). However, critical questions remain on estimating the complex interconnected linkages between the socioeconomic benefits associated with goats and understanding the drivers of variation across households in drawing benefits from goat production.

Livestock production in Botswana primarily consists of commercial, mixed smallholder communal and traditional systems (Burgess, 2006). Amongst them, communal goat production systems operate with a mean household holding of 21 goats (Statistics Botswana, 2017). Goats are extensively managed, mostly in communal grazing areas during the day and kraaled (enclosed) at night (Burgess, 2006). The performance and outcomes of goat production systems vary greatly but are generally poor, with Statistics Botswana (2017) reporting low off-take rates of 7.3% and a high mortality rate of 22.3%. Across SSA, goat mortality has been predominantly attributed to wild animal and dog attacks and disease (Airs et al., 2023; Moagabo & Baipoledi, 2008).

Poverty in Botswana has been steadily reducing, from a rate of 42% in 1985 down to 13.5% in 2023 (World Bank, 2024). However, on the Global Food Security Index 2022, Botswana ranks 87th (of 113) (The Economist, 2022) and undernourishment remains prevalent at 24% (FAO, 2022). Previous research has shown how livestock may facilitate households in diversifying their food and income sources (Acosta et al., 2024; Murendo et al., 2019). Romeo et al. (2016) reported, in Kenya, that investment in small livestock assets (including goats), especially when driving diversification, positively contributes to household income and food security. Therefore, more productive and resilient livestock systems may hold the potential to close the gaps in poverty, food security, and nutrition. However, disease, climate, nutrition, and market access have been identified as factors that may limit the development of livestock production systems (Kahi & Wasike, 2019; Rumosa Gwaze et al., 2008). It is therefore pertinent that we understand the nature of these dynamics. To this end, the study aimed to: (1) identify relationships between goat ownership and household financial and food security, (2) identify barriers that are limiting to smallholder goat enterprises and the opportunities for overcoming these.

## Methods

Data collection was approved by research permit number ENT 8/36/4 XXXX II (5) by the Ministry of Environment, Natural Resources Conservation and Tourism. Participation was optional and all participants gave informed consent via forms in the language of their choosing and those who had limited literacy were read the consent form in their local language.

### Sampling and site description

**Fig. 1:**
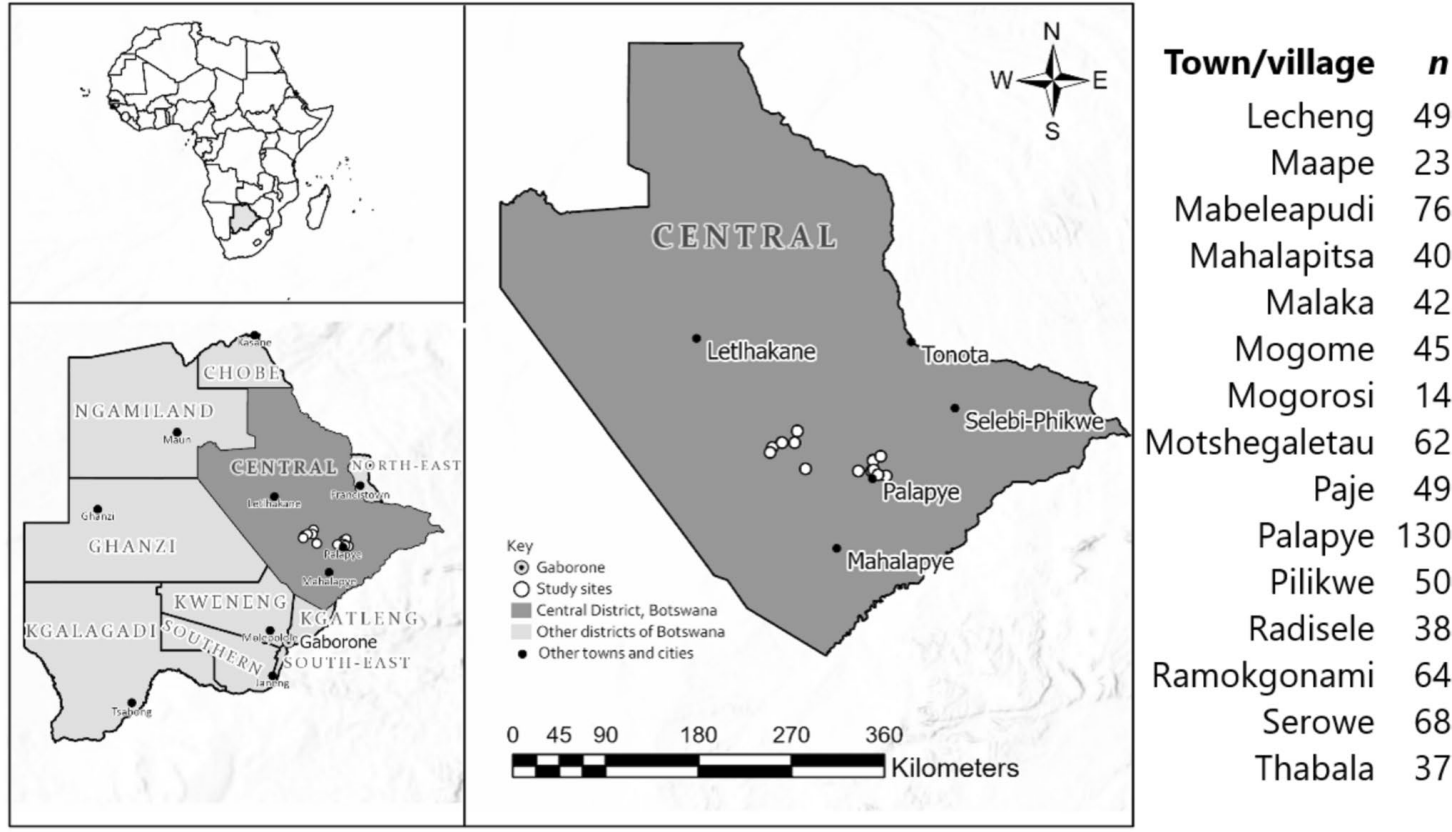
Map of region and study sites as white points and major towns and cities as black points. Right, list of towns/villages covered by the survey and number (*n*) of responses from that location for the cross-sectional survey

Surveys were conducted throughout November and December 2019, across the Central District of Botswana, covering 14 villages (Fig. 1) by 6 enumerators. Surveys were conducted in two stages, with a general, short but fully structured, cross-sectional survey (*n* = 787) followed by a more detailed semi-structured survey for a subset (*n* = 44) of goat smallholders. Access to villages was guided by personnel from the Department of Animal Production and Health (DAPH) (small-stock production improvement section), under the Ministry of Agriculture in Botswana. Depending on the distribution of the households, enumerators were distributed in all populated areas of the village and would start from the edge of a block of houses or settlements. Households were randomised by skipping 5 households (where households were spaced out) or ten households (where they were densely populated); then selecting the sixth or the eleventh household, respectively. Where the household head or adult family member capable of representing the head (described as the respondent in this study) was not available at the selected household, the next available was selected without further skipping. Within that process, where a respondent indicated that he/she had at least ten goats, a more detailed semi-structured questionnaire was then used. The detailed survey included additional questions on aspects of goat husbandry. Electronic tablets were used for data collection. The questionnaire was deployed using KoboCollect and responses were instantly entered and saved by enumerators. For elderly and/or less literate respondents, the questions were posed in an appropriate local language, i.e. Setswana. The surveys were designed as follows (full version available in Appendix A):

- **Demographic:** Age, gender, location, educational attainment, household size.
- **Food & financial security:** Income sources, financial freedom, concern about food, number of meals per day, and ownership of goods (e.g. car, mobile phone).
- **Livestock**: Type and numbers of livestock owned, ranking of importance of each.
- **Goats:** Husbandry/housing, feed supplementation, mortality, herd composition, offtake.
- **Additional questions (detailed survey):** Contribution of goats to household income, desirable goat traits, specific health and mortality cause specifics, availability, use and affordability of veterinary medicines and services.

The Central District of Botswana is characterised by hard-velt open bush savannah on a mix of sandy (sandveld) and rocky (hardveld) soils. The region experiences distinct seasonality with low rainfall and cool temperatures during the dry season (April to October) and high rainfall, temperatures, and humidity during the wet season (November to March). This seasonality impacts forage availability as herbaceous species reduce in abundance during the dry seasons, with mainly browse species persisting (Cooke et al., 2024c).

### Data analysis

Data was analysed in two parts, i.e. firstly, those from the cross-sectional survey were analysed with a particular emphasis on goat and livestock ownership, focusing on overarching characteristics and tendencies of rural households in the region (including non-goat owners). Thereupon, results from the second-stage survey were analysed to elucidate more nuanced elements of goat ownership, particularly barriers and opportunities for goat production.

Statistical tests comparing factors across goat ownership levels based on the number of animals in each herd (1–10, 11–20, > 20) were compared using Chi-squared tests if the data was binary (e.g., use of anthelmintics) or Kruskal–Wallis tests if data was continuous, count, or ordinal (e.g., off-take rates). A Chi-squared test was also used to identify differences in supplementation use based on the ranking of agriculture towards household income.

Food security, measured by the number of meals per day for adults and children, was compared to goat ownership (binary), using a two-stage least-squares regression (2SLS). The importance of agriculture to household income was used as an instrumental variable for goat ownership because it was strongly associated with goat ownership (F = 53.21, *p* < 0.001), exceeding the typical threshold of *F* ≥ 10. This was not expected to directly influence meals per day except through goat ownership. The Wu–Hausman test confirmed the endogeneity of goat ownership (F = 19.44, *p* < 0.001), supporting the instrumental variable approach. Financial security, measured by the extent to which income meets needs, was compared against the use of supplemental goat feeds using a Chi-squared test.

A conditional beta-regression model was used to compare approaches to the use of feed supplements. Beta regression was chosen over other models (such as a Generalized Linear Mixed Model) because the mortality data were expressed as continuous proportions (between 0 and 1) rather than raw counts. Beta regression is well suited to modelling proportional data and can effectively handle skewness and heterogeneity. The model included mortality (as the percentage of goat deaths in relation to the total number of goats in the previous 12 months) as the dependent variable. Supplementation with foliage, food waste, grain, and crop residues were included as binary independent variables. However, the inclusion of these supplements in the model was conditional on the value of “grazing only” of the goats, i.e. when they received no supplements, the other model terms were excluded for that record.

## Results

### Cross-sectional survey

#### Sample summary

The sample was female-dominated (72%) and weighted towards older respondents, of whom 32% were over the age of 60, while bearing in mind a life expectancy of ~ 66 years in Botswana, (Table 1). Educational attainment was mixed, and the majority of respondents had at least some degree of formalised school education.

**Table 1:** Summary of survey respondent characteristics 1) all respondents 2) respondents who reported to own any form of livestock, and 3) respondents who specifically owned goats

		All respondents (*n* = 787)	Livestock owning respondents (*n* = 508)	Goat owning respondents (*n* = 442)
Gender	Female	72%	68%	67%
	Male	28%	32%	33%
	< 30	17%	18%	17%
	30–39	16%	17%	17%
Age	40–49	18%	14%	14%
	50–59	17%	18%	17%
	> 60	32%	34%	35%
	None	22%	21%	22%
	Primary	33%	35%	35%
	Form 4–5 (senior)	12%	12%	13%
Educational attainment	Junior certificate	27%	26%	25%
	Tertiary	5%	4%	3%
	Vocational	2%	2%	2%

**Fig. 2:**
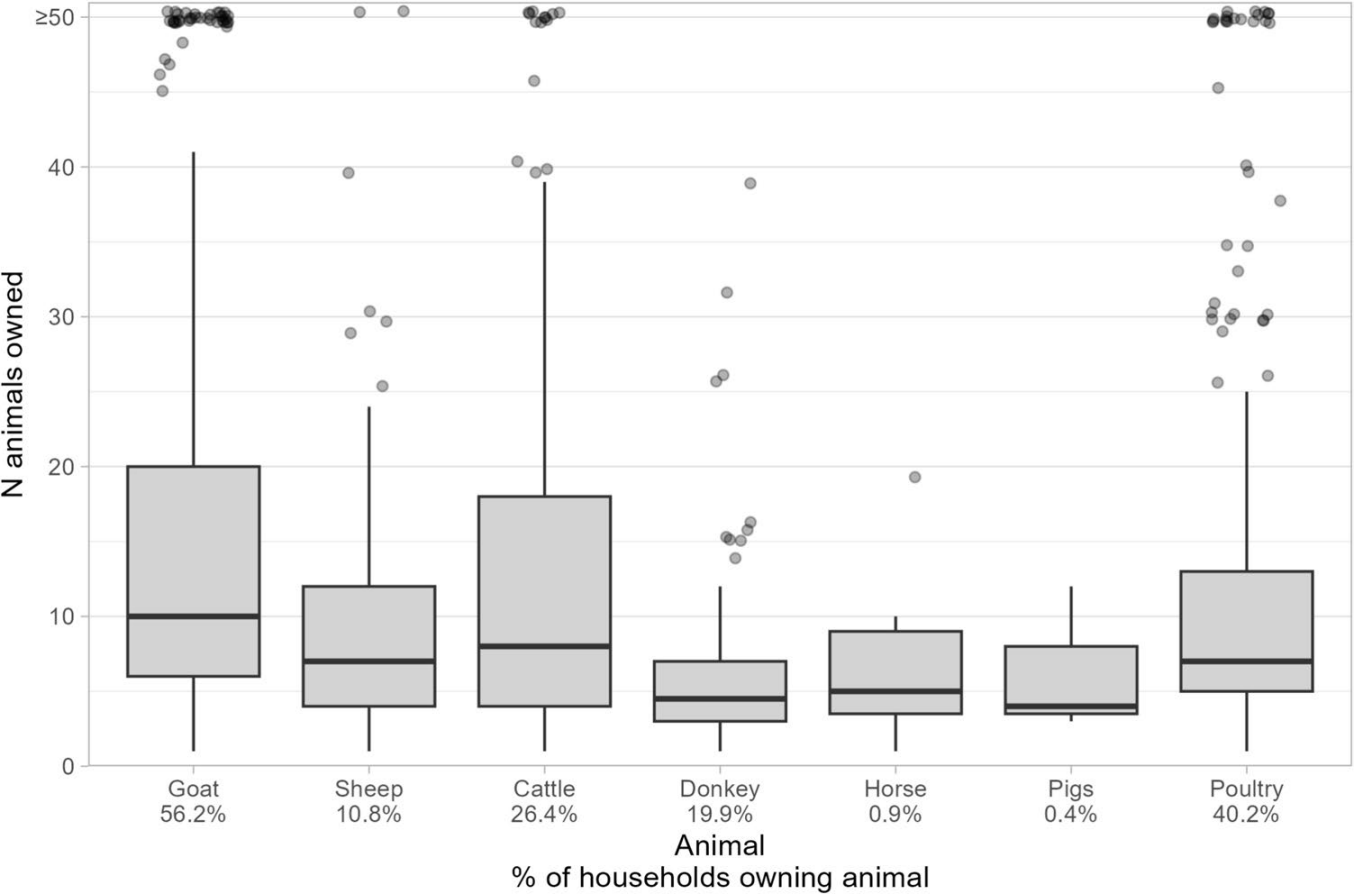
Number of each animal owned for households owning one or more of said animal. The percentage of respondent households that own one or more of a given animal is provided on the x-axis, under the animal’s name. Please note that values clustered at the top of the y-axis represent households that own 50 or more of a given animal

Of the 787 respondents, 65% owned at least one species of livestock (Fig. 2) of which goats were the most commonly owned, with 56% (442) owning at least one goat. Poultry ownership was also relatively common (40%), though with a large variation in flock sizes. Furthermore, goat ownership was strongly associated with ownership of other livestock or household assets. For instance, goat owners possessed an average of 5.5 (± 2.1 SE) cattle compared to an average of 1.3 (± 1.6 SE) for those without goats.

Adults and children typically consumed two meals per day (Table 2). However, while adults in 21% of households typically consumed just one meal per day, this was only true for children in 16% of households. A large majority of respondents (76%) reported having been concerned about the household having sufficient food in the past week. Goat-owning households typically consumed more meals per day than non-goat-owning households (Table 2). This trend was true for both adults (means 2.2 vs 2.0, *t* = 5.039, *p* < 0.001) and children (means 2.1 vs 1.9, *t* = 4.481, *p* < 0.001). Importantly, as goat holding increased, off-take increased from 6 to 12% and worry about food decreased from 79 to 65% of the households involved.

**Table 2:** Responses to food security questions for 1) all respondents 2) respondents who reported to own any form of livestock, and 3) respondents who specifically own goats

Meals per day		All respondents (*n* = 787)	Livestock owning respondents (*n* = 508)	Goat owning respondents (*n* = 442)
Adults	1	21%	17%	17%
	2	51%	51%	50%
	3	28%	31%	31%
	≥ 4	1%	1%	1%
Children	1	16%	13%	13%
	2	44%	43%	44%
	3	37%	40%	40%
	≥ 4	3%	4%	5%
Concerns about food in the past week	Yes	76%	74%	74%
	No	25%	26%	26%

The most common primary sources of cash income were agriculture and unrelated benefits (including pensions). Cash income data was skewed towards benefits by the high representation of respondents aged over 60 in the sample. Within agriculture, livestock owners were more likely to own any given asset than non-livestock owners. The trends observed between goat ownership and food security were not observed between goat ownership and financial security (Table 3) Whilst households owning goats did have slightly better financial security (as measured by income sufficiency), this difference was not statistically significant (*t* = −0.441, *p* = 0.659).

**Table 3:** Responses to financial security and income questions for 1) all respondents 2) respondents who reported to own any form of livestock, and 3) respondents who specifically own goats

		All respondents (*n* = 787)	Livestock owning respondents (*n* = 508)	Goat owning respondents (*n* = 442)
	Agriculture	20%	27%	29%
	Wage	31%	28%	27%
	Business	14%	13%	13%
Main income source	Remittance/gift	8%	7%	7%
	Pension/benefit	25%	22%	21%
	Impelegen/public service	2%	2%	2%
	Self-employed	0%	0%	0%
	Insufficient, borrows	29%	31%	26%
Income sufficiency	Insufficient, uses savings	4%	4%	3%
	Meets expenses	49%	48%	52%
	Allows saving	18%	18%	19%
	Donkey/cart	29%	39%	39%
	Bicycle	12%	15%	16%
	Motor-bike	1%	1%	1%
	Wheel-barrow	51%	58%	58%
Ownership of asset	Sewing machine	5%	5%	5%
	Fridge	44%	45%	46%
	Radio	57%	59%	58%
	TV	48%	50%	50%
	Mobile phone	87%	88%	88%
	Car	9%	9%	10%

#### Goat husbandry

**Fig. 3:**
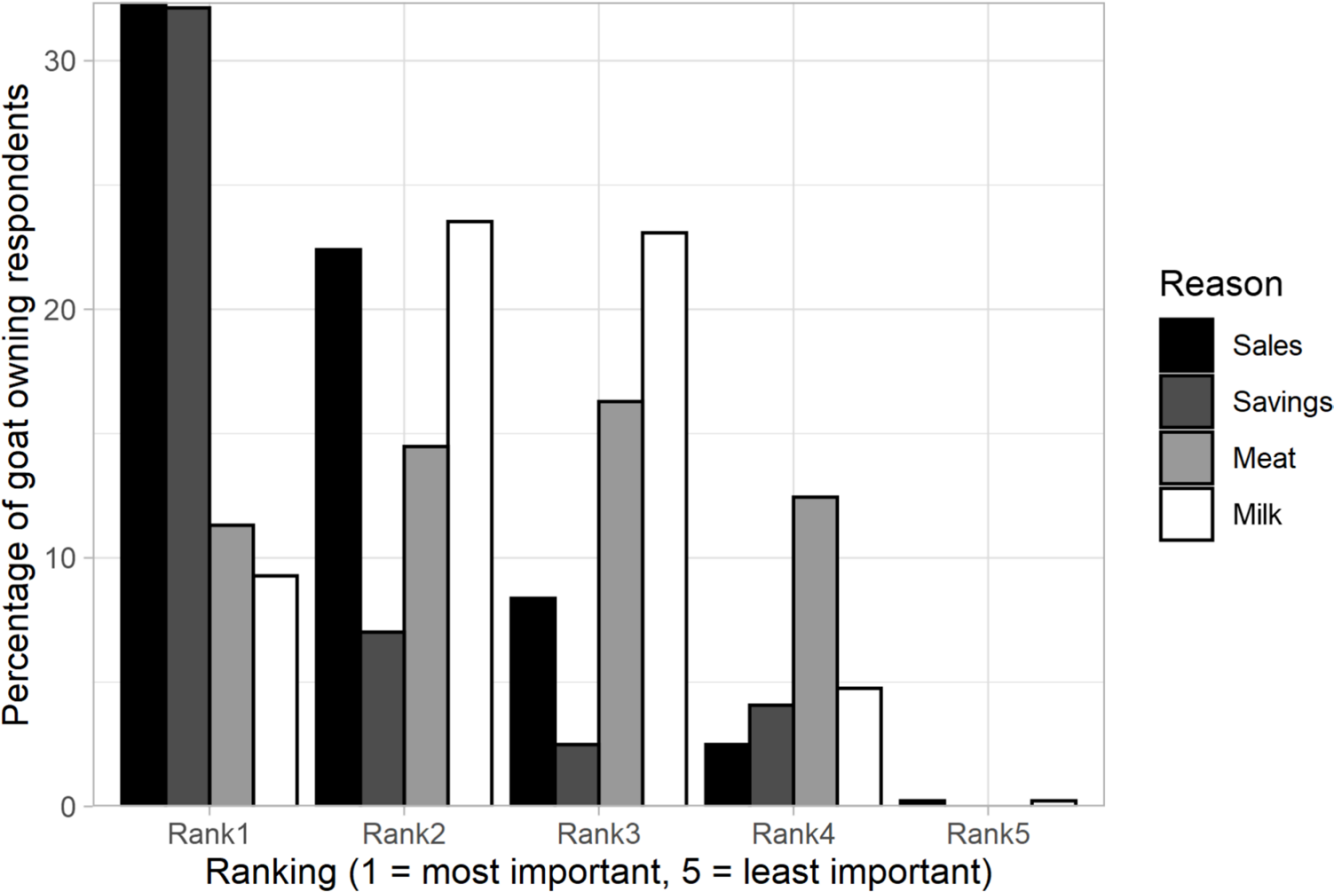
Reasons for goat ownership ranked in terms of perceived importance

Reasons given for goat ownership were not only diverse, but most respondents gave multiple reasons (Fig. 3). The most cited reason was as a source of cash (38%) or insurance, ascribed to either cash or nutritional (38%). Provision of milk and meat were also important reasons, but typically secondary to financial motivations.

Adult goats predominantly free-grazed during the day but were brought into a kraal/boma (enclosure) at night, both during the dry season (98%) and the wet season (98%). Whilst this was also the most common management strategy for goat kids, it was less prevalent at 60% during the dry season and 61% during the wet season.

**Fig. 4:**
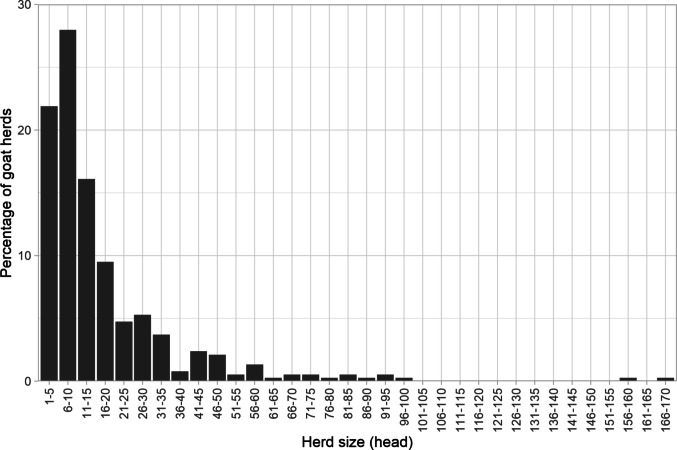
Distribution of goat herd sizes

The local Tswana (or mixed) goat breed was the most common (97%), with a mean herd size was 17 and a median of 11 (Fig. 4). However, the variation in herd size was large (s.d. = 20.0) with a strong positive skew and the most common herd size grouping being 6–10 goats. Herds were typically composed of a majority of adult females (61% of herds), with kids being the next most common (Table 4).

**Table 4:** Goat herd structure represented by mean and median numbers of different types of goats owned

	Mean	Median	s.d
Adult female	9.6	6	11.5
Castrated male	2.5	1	4.5
Adult buck	0.8	0	1.3
Suckling	0.2	0	2.7
Kid (< 1 yr)	4.2	3	5.9

A large majority (96%) of goat owners stated that they wished to increase their herd size. However, there were several factors cited as being barriers to achieving this, the most common being availability of cash (56%), followed by concerns about feed availability (16%), disease (14%), water availability (6%), labour demands (4%), and dog attacks (3%). Several reasons were also given by respondents under an option of “other”, and included: theft, reproductive failure, availability of breeding stock, space, traffic collisions (for those close to highways), or being new to goat ownership (all < 5%).

**Fig. 5:**
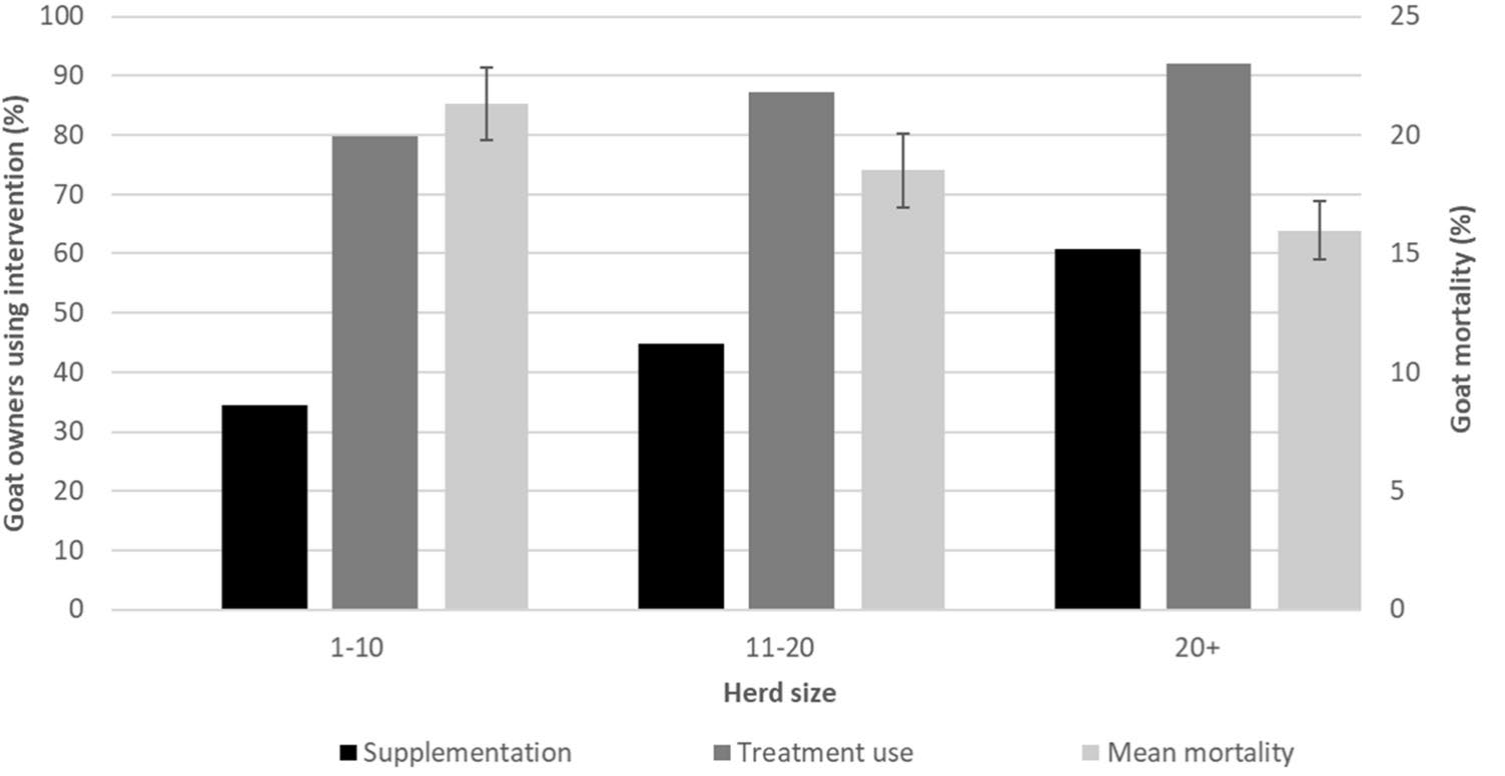
Use of supplementation, use of antiparasitic (endoparasite and ectoparasite) treatments, and mortality, across different herd sizes. Black bars represent the % of goat owners using supplementation (primary y-axis). Mid-grey bars represent the % of owners using veterinary treatments (primary y-axis). Light-grey bar represents goat mortality with error bars representing standard error (secondary y-axis)

Larger herd sizes were associated with a greater use of feed supplementation and treatments for parasites, and lower mortality rates of 15.8% compared to 21.3% for farmers with smaller herds (Fig. 5). For farmers with a herd size larger than 20, the use of supplemental feeds was almost double that of those with a herd size of 1–10. The percentage of owners reporting the use of veterinary treatments was high regardless of herd size, but more so with greater herd sizes.

**Fig. 6:**
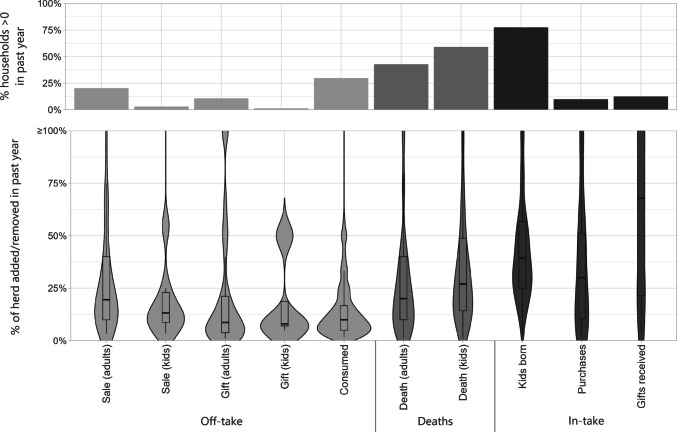
Addition/removal of goats to the herd over the past 12 months, split by types of off-take, deaths, and in-take. Top: The % of households that reported 1 or more goat, over the past 12 months, added or lost for any reason listed. Bottom: Of those households, the % of the herd that was added or removed from the herd, as a proportion of their current herd size (thus value could exceed 100%). Note: Ceremonial use was not plotted on the graph as only one household reported such use (1 goat used, current herd size of 6)

Average off-take rates were low, with the most common reason being the sale of adult goats. However, there was a large variation in off-take rates between herds (Fig. 6). Death rates were high, especially for kids; in the year previous to the survey, 39% of households had experienced at least one or more unexpected goat deaths in their herd, with the mean number being 5 deaths. Over the same period, 72% of respondents reported at least one kid being born, with the median number of births being 3.

The majority (53%) of herds relied solely on grazing for feed, but supplementation was also common especially through the use not only of crop residues (20%) and foliage (15%), but also grain (10%), and food waste (8%).

Even though financial security and the use of supplemental goat feeds appeared to be positively associated, this was not statistically significant (ꭓ^2^ = 5.899, *p* = 0.117). Those who stated that agriculture was their primary source of income were more likely to use supplements (54%) than those who did not (30%) (ꭓ^2^ = 21.797, *p* < 0.001).

**Fig. 7:**
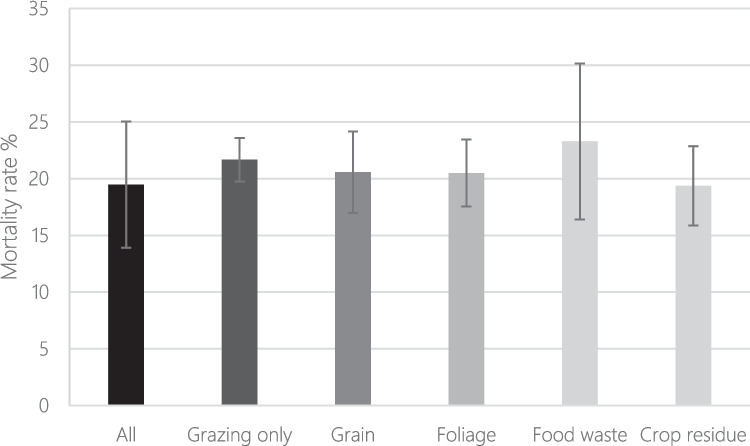
Mortality rate (% of goats owned over past 12 months) separated by the use of different supplemental feeds. Error bars represent standard error

Although the highest observed mortality rate was in herds receiving food waste, this only included 11 respondents, and variation was high (Fig. 7). Conditional beta-regression results, which accounted for exclusive grazing, showed mortality differed across herd management. Goats that exclusively grazed had significantly higher mortality than those receiving supplements (grazing: estimate = 0.568, z = 2.905, p = 0.004). Mortality tended to be higher with grain (estimate = 0.328), foliage (0.356), food waste (0.293), and crop residues (0.257) but these were not statistically significant (p > 0.06). Model precision was moderate (phi = 7.379, p < 0.001), supporting the reliability of the fit. Overall, exclusive grazing was associated with higher mortality, however, the high mortality in the food waste group suggests potential, but unconfirmed, risk from this supplement type.

Ectoparasite control using chemical treatments (e.g. dips, sprays, or paraffin) was common (Table 5). Endoparasite control was moderately common, especially using anthelmintic drugs.

**Table 5:** Use of different control strategies for ectoparasites and endoparasites of goats. ‘Chemical’ refers to the use of non-pharmaceutical chemicals, such as paraffin

	Ectoparasitic	Endoparasitic
Drugs	2%	57%
Chemical	95%	15%
Nutraceutical/bioactive plants	1%	3%
Manual removal	2%	n/a

### Semi-structured survey

#### Contribution to household income and food security

Out of 44 respondents, 16 were unable to specify their income from goats as a percentage of household income. Six respondents gave a value of 0%, with another two stating they do not normally sell. Whilst one farmer stated that income from goats was low because they were unable to find buyers, another stated they were able to build a house from goat sales. From the remaining 18 farmers who did provide a value above 0%, the mean contribution of goats to household income was 57% (median 58%, s.d. 17.9).

When asked the importance of goat production to household food security, 73% of respondents stated it was ‘extremely important’ and 18% stated it was ‘very important’. When asked a similar question, about the importance of goat production towards cash income, 64% reported it to be ‘extremely important’ and 25% ‘very important’.

#### Health & productivity

When asked to rate the health of their herd (compared to other local herds) from 1–5 (1 = less healthy, 2 = somewhat less healthy, 3 = similar, 4 = somewhat more healthy, 5 = much more healthy), most participants rated herd health as 4 (Table 6). Farmers who responded that their animals were healthier than others locally (4 and 5), predominantly attributed it to knowledge, management, use of drugs/vaccines, grazing, and access to water. For those who gave lower scores of 2 (somewhat less health) and 3 (similar), this was mostly attributed to a lack of funds for feed and treatments, or to drought. Self-assessed scores for productivity were positively associated with those for health (Table 6). Factors considered indicative of good productivity were given to be good meat sales and management skills. Reasons for poor productivity related to health and focussed on droughts, limited access to medicines, and consequent disease.

**Table 6:** Self-assessed herd health and productivity ratings from 1 (worst) to 5 (best). Numbers inside unshaded squares signify the number of respondents who answered in the combination of health and productivity scores

		Health				
		1	2	3	4	5
	1	0	1	1	0	0
	2	0	6	0	2	0
Productivity	3	0	1	4	4	0
	4	0	1	1	11	2
	5	0	0	0	1	9

Despite the generally high self-assessed scores for health, lameness and ill-health in kids were widely reported with 84% stating that lameness was a problem for their goat kids and 84% (with all but two of those respondents overlapping entirely) reporting that ‘sickness’ had been an issue with kids. Causes of sickness were relatively broad and included internal parasites/worms (8), pasteurellosis (7), heartwater and ectoparasitoses (7), foot-rot (6), anaemia, heart and blood diseases combined (5).

The traits most sought after in goats could be characterised into four main categories of themes, namely fertility (mentioned 15 times, particularly twinning), weight gain, growth, feeding, and size were collectively mentioned 13 times. General health was mentioned 10 times, meat quality 7 times, and milk production once. Profitability was only mentioned twice, but this may be due to poor interpretations of the question posed.

When asked about anthelmintic (dewormer) use, 11 respondents stated that they used terramycin (an antibiotic) to this end. Only four respondents specified the use of a specific anthelmintic, i.e. ivermectin or albendazole. Only six respondents stated animals were weighed to determine dosage and the majority (30) stated that administration was by injection. The most common source of anthelmintic treatments was the Botswana Agricultural Marketing Board (BAMB).

Faecal egg counts (FEC) as a means of detecting endoparasite infections were relatively uncommon with 32% of respondents stating that these had been performed on their animals previously. This is possibly an issue of availability of equipment or services for FEC, with 59% of respondents citing that it was unaffordable and respondents stating that they would need to travel to the main towns in the area (Palapye or Serowe), and/or a mean distance of 15 km, whilst bearing in mind that only two respondents owned cars.

#### Grazing management

Rotational grazing was employed by a small majority of participants (52%). The reasons given for this were mainly to avoid overgrazing and to ensure goats had access to sufficient pasture. However, five stated they moved goats to avoid them consuming crops (either owned by them or others). Those who gave a reason for not rotationally grazing mostly stated that this was because they had access to extensive or communal grazing lands.

All smallholders released their animals for grazing daily during both the dry and wet seasons with a respective mean grazing period of 8.7 h per day in the wet season (median 8, s.d. 1.6) and 7.4 h in the dry season (median 8, s.d. 2.5).

## Discussion

Goat ownership was positively associated with household food security. Differences in food security between goat and non-goat-owning households were predominantly assessed through number of meals per day and compared between adults and children. This is indicative of parents foregoing food for themselves, in order for their children to be able to eat. Worry about food in the week of the survey was not different between households that owned or did not own goats, but those with higher numbers of goats reported being less worried.

While financial security between goat owning and non-goat owning households did not significantly differ, farmers reported a 57% contribution of goat enterprise to household income. This is linked to the critical role of goats being kept as insurance in smallholder farmers, where they are sold to cover urgent cash needs. In this current study, farmers cited cash or insurance as the main reasons for keeping goats (Fig. 3). Indeed, in contexts where formal financial and credit markets are poorly developed, capital and savings are invested in livestock as future financial guarantees or insurance for unexpected needs (Wodajo et al., 2020; Kaumbata et al., 2020; Byaruhanga et al., 2015). Goats thus provide an insurance and credit livelihood buffer in emergency periods (Airs et al., 2023; Joy et al., 2020). It thus follows that where farmers frequently sell goats for income, they may consider themselves less financially secure as they are more reliant on converting their savings/insurance (goats) into cash to cover needs rather than alternative assets or financial sources.

Goat-owning households with larger herds tended to possess other forms of livestock and durable assets (such as cattle, wheelbarrows, and mobile phones), indicating that goat ownership is often part of a broader livelihood strategy. This raises the possibility that goats both contribute to, and are a reflection of, household wealth, supporting their role within well-balanced household asset portfolios. Reflective of this was the fact that households with larger herds were more likely to invest in supplemental feeding and veterinary care, both of which were associated with reduced mortality and increased productivity.

The relationship between financial and food security is reciprocal. Capital investment is necessary to maintain and improve goat enterprises, generating income. Increased income allows households to better secure food through direct consumption or market purchases. Thus, goats can function both as a source of immediate food security and as an economic asset that supports longer-term livelihood stability.

### Barriers and opportunities

Several barriers to goat enterprises were identified, each presenting an opportunity for investment and intervention. The following subsections present a discussion of the key identified barriers.

#### Capital investment and turnover

Despite financial reasons, particularly cash sales, being the most common motivation for rearing goats, goat sales were reported to be low in number, with most smallholders selling none in the previous year. This limited turnover may mean that goat enterprise could inadvertently tie up assets. Manirakiza et al. (2020) found that farmers with smaller herds derived insignificant income from their goats, with 45% not engaging with markets at all. One-third of smallholders in the present study stated that their income was insufficient to meet their expenses, yet nearly all expressed a desire to increase their herd size – though the reasons for this are not defined, they are likely driven by both cultural and economic factors. These results highlight the financial barriers that smallholders face, with many situated in a scenario in which they want more goats to increase their income but lack the income for that investment.

There are opportunities to overcome this barrier through directly supporting individuals or through cooperative programmes. This could take the form of grants, loans, and other financial instruments (at individual or group level) or possibly the shared ownership and management of resources where it is more feasible or efficient to do so (e.g. machinery). For example, in the off-take model in Kenya, off-takers provide access to insurance and credit services facilitated financial inclusion for pastoral goat keepers (Otieno, 2025). Community-based goat breeding programs have demonstrated potential to reduce barriers associated with feed resources and pasture management (Kaumbata et al., 2021).

Helping farmers to increase the sizes of their herds could be beneficial for smallholder farmers’ household income and food security. Such farmers strategically increase the proportion of goats marketed as herd size increases, to support livelihoods, food security and other agricultural activities. Goats contribute to food security directly through meat and milk products, and indirectly through income from goat marketing (FAO, 2015; Idamokoro et al., 2019; Kumar et al., 2010). Kaumbata et al. (2020) indicated that most smallholder farmers sold goats strategically during the dry season to stock food reserves and purchase agricultural inputs, thus evidencing the synergy between goats, food security and smallholder farmer incomes.

#### Supplementation

Farmers with larger herds were most likely to use feed supplements. Herds provided with supplemental feed typically had reduced mortality. The exception being those given food waste as supplemental feed (notably only *n* = 11), which had the highest mortality rate; although the reason for this is unclear it may be through inappropriate nutrition, degradation/fermentation and, contamination with food-borne pathogens. There is a cost to acquiring supplements, either financial if they are purchased, or to labour input if they are harvested. The use of naturally available vegetation for supplementation, such as browse plants (i.e. *Terminalia* spp., *Acacia* spp.), may offer an opportunity to overcome the financial barrier of purchasing supplements (Cooke et al., 2024b). There may also be a factor of prioritisation, with those ranking agriculture as less important to their household income being less likely to use supplementation.

Although goats are reported to survive on low planes of nutrition (Joy et al., 2020; Sebei et al., 2004; Visser, 2019), this can limit resilience to stressors. For instance, an optimal energy supply is important for goats to withstand high heat stress (Joy et al., 2020) and well-nourished goats tend to be more tolerant to internal parasites (Hoste et al., 2005). Thus, poor nutrition and lack of supplementation in extensively reared goats negatively impacts productive performance, for instance compromising host resistance to diseases.

The potential nutraceutical role of supplementation must also be considered. Plants available in the region such as *Boscia* spp., *Terminalia* spp., *Acacia* spp., *Viscum* spp., and others have contain plant secondary metabolites that can improve host responses to a range pathogens (Badar et al., 2011; Waterman et al., 2010; Zabré et al., 2017). Several studies have also indicated the benefits of tannin-rich tree foliage for ruminant nutrition and integrated nematode control (Bakare et al., 2020; Hoste & Torres-Acosta, 2011; Hoste et al., 2006; Machekano et al., 2025).

#### Veterinary care

Results from the semi-structured survey revealed two primary barriers to access to veterinary services. The first is the cost of accessing veterinary services, relative to income, including consultation, medicine, diagnostic, and travel costs. The second barrier is the distances between farmers and veterinarians or LIMID (Livestock Management and Infrastructure Development) offices. Most smallholders do not own cars, typically living > 15 km away from their nearest veterinary service provider, as also reported elsewhere (McKune et al., 2021). LIMID or veterinary officers are also constrained by resources such as fuel, vehicles and personnel (Binge et al., 2019).

High goat mortalities have been reported among resource-poor smallholder communities in SSA. Mortality rates 13% has been reported in Ethiopia (Alemu et al., 2019), 40% in South Africa (Slayi et al., 2014), 35% in Malawi (Kaumbata et al., 2020; Nandolo et al., 2016) and 18.3–27.2% in Burundi. We did not have sufficient data to investigate further the causes of mortality, or identify nutrition and disease related mortality causes, which are often intricately linked. Mortality was therefore associated with a combination of disease, nutrition, and dog/wild animal attacks, causes which as reported in other studies elsewhere (Slayi et al., 2014). Future studies should thus disentangle disease, nutrition, and predation to pinpoint the most plausible causes of kid mortality. Most resource-constrained smallholder farmers also lack the resources for nutritional supplementation and veterinary care for their goats, hence disease-nutrition related mortality remains an important and commonly experienced challenge (Mdladla et al., 2017; Monau et al., 2017, 2020; Visser, 2019). Indeed, Mdladla et al. (2017) and Manirakiza et al. (2020) confirmed the above, particularly for smallholder farmers with small herds, who spend negligible amounts of money on veterinary drugs due to financial resource limitations and the high costs of anthelmintics (Sanhokwe et al., 2016; Walker et al., 2015), which is often exacerbated by the inaccessibility of the anthelmintics due barriers to accessing veterinary services.

Limited access to diagnostics or training means that targeted selective treatment (TST) is not widely practised in the study area. This approach could attenuate treatment costs and the risk of the development of anthelmintic resistance (Kenyon et al., 2009), as well as the negative ecological impacts (Cooke et al., 2017; Sands et al., 2018). The use of TST has been shown to yield positive health and economic outcomes for smallholder goat farmers in Botswana (Walker et al., 2015). To this end, methods such as the Five Point Check^©^ and FAMACHA scoring can be effective tools for farmers (Bath & van Wyk, 2009; Olah et al., 2015; van Wyk & Bath, 2002). In the short to mid-term, at least, training in such techniques is likely to be more feasible than improving the access to and affordability of veterinary care.

Interesting, terramycin (an antibiotic) was reported used for helminth control for 11/44 farmers in the semi-structured survey. This could be due to, among others, the communication of symptoms between farmers and vets leading to a presumptive diagnosis and inappropriate treatment, or to substitution of anthelmintics with antibiotics based on cost or availability. This could pose short-term risks to the goats, which then do not receive appropriate anthelmintic medication, and longer-term risks to the wider goat population as this may drive the development of antibiotic resistance. Evidence elsewhere has shown similar patterns in agricultural systems in the region, for example the use of fungicides an nematicides against insect pests of crops (Machekano et al., 2019; Obopile et al., 2008).

This gap in veterinary care could be filled by training and deploying veterinary paraprofessionals (Kelly et al., 2022). This could help to increase access to veterinary knowledge and skills from a community driven approach, also increasing trust in veterinary services (as reported to be a constraint by Namatovu et al., 2025). This also provides an opportunity to empower women within the sector, tackling an additional barrier of gender by providing unique business opportunities and valued skills (Kelly & Herrington, 2025).

#### Age and gender

The high proportion of female respondents aligns with previous findings regarding gender roles and goat ownership patterns skewed towards women in rural households (e.g. 66% by Moepeng & Tisdell, 2008). This highlights a paradox because community leadership and land tenureship are typically male-dominated, with Statistics Botswana (2019) reporting that 37% of ‘agricultural holders’ are female. This may prevent women from making decisions regarding land use and how livestock are managed. As women and youths largely assume ownership and greater participation in goat rearing than cattle, decision-making dominance by men in land and livestock issues has implications for their contribution to agriculture, food security, economic autonomy and household decision-making (Njuki & Sanginga, 2013; Bettencourt et al., 2015, Wodajo et al., 2020). This may also exacerbate other barriers associated with financial incapacity as women may be limited in their ability to access resources to support agricultural activity.

Access to financial instruments, technology, and decision-making powers by women may also be barriers to investment and development in livestock systems (Monau et al., 2020; Waithanji et al., 2015). Increasing access to resources and decision-making power could provide a synergistic opportunity for the elevation of the socio-economic status of female smallholders whilst also improving goat health and productivity (Bain et al., 2020). Acosta et al. (2024) reported a positive association of livestock ownership with household income and food consumption, however, this effect was strongest in female-headed households. As mentioned previously, the training of veterinary paraprofessionals could be a mechanism for female empowerment in the sector. Farnworth et al. (2015) and Johnson et al. (2015) reported that women who received training were consulted more by men in household decisions, and contributed more to the household livelihood processes through their human capital.

Farmers aged 60 and above represented 35% of goat-owning participants in the cross-sectional survey and 39% in the semi-structured survey, which broadly reflects values reported elsewhere, where 35% of goats in the country were owned/managed by the 65 + age group in Botswana (Statistics Botswana, 2019). Consequently, this may present a challenge due to the lower uptake of new technology by older individuals, reduced entrepreneurship (Syed et al., 2024), and the possibility of poor health and fitness limiting the capacity to undertake labour-intensive activities.

### Regional applicability

The results presented here are broadly applicable to other countries within the southern Africa region and, to a lesser extent, wider SSA. Food insecurity and financial insecurity are common issues across rural SSA (Giller, 2020). The risks to livestock are also comparable, with malnutrition, disease, and animal attacks driving relatively high mortality in many communities. Climate does vary but has broad overarching characteristics of distinct seasonality with risks of drought and extreme temperatures (WMO, 2025). However, in applying these results to other contexts, it is important to be cognisant of climatic, political, cultural, and economic differences. For example, goat sales in Botswana are typically conducted on the basis of liveweight, whereas in Malawi a price-per-head system is more commonly used. These different models may be sufficient to impact the strategies that smallholders employ within their systems.

### Climate change

Under the pressures of climate change, the challenges faced by smallholder goat producers in Botswana are expected to intensify. Changes in rainfall patterns and consequent impacts on biomass availability represent some of the most severe threats to natural capital within these systems (Assan, 2022). Such shifts are likely to exacerbate existing nutritional feed gaps (Cooke et al., 2024a). Whilst supplementation can be used as a mitigation strategy, supplementation already represents a barrier for many due to cost and accessibility, and climate change may further amplify this challenge by increasing demand, whilst exposing farmers to market pressures and supply chain vulnerabilities.

Climate change is expected to impact goat health through reduced water availability, heat stress, and shifting disease epidemiology. For instance, Olwoch et al. (2008) predict that the incidence of Theileriosis (East Coast Fever) could rise in Botswana and the broader region under climate change. Increasing disease prevalence could escalate the need for veterinary services – exacerbating the barrier of limited veterinary service access.

### Study limitations

Whilst the gender imbalance in the sample is similar to elsewhere (e.g. Moepeng & Tisdell, 2008), it may still represent a bias. The semi-structured survey had 44 respondents, which is a relatively small sample size. However, this is not entirely unusual for more detailed components of such work and also given the small population size of the sampling location. For several topics, additional information from farmers would have facilitated a stronger understanding of the situation. For example, when considering terramycin use, a follow up discussion to discern the reasons for that would have been valuable – investigating this and other findings in more detail is of interest for further study. This study focussed at the smallholder level, but there are undoubtedly barriers beyond the results and scope of this study, such as climate change. Therefore, future work to research and address this issue should not limit itself to the barriers identified here.

## Conclusion

This study explores the contribution of goat enterprise to rural livelihoods in Botswana. Whilst goat production can be an income-generating enterprise for many, they predominantly function as livelihood buffers. Goat ownership was associated with improved household food security, but variation in outcomes was driven by herd size and resource constraints.

Goats are a flexible asset that can help households absorb shocks, manage risk, and stabilise food and finances, against a backdrop of insecurity. However, several barriers to goat enterprise development were identified, namely capital investment and turnover, nutritional supplementation, veterinary care and access, and age and gender. These barriers are interwoven as part of complex and dynamic livelihood strategies, whilst also being impacted by externalities such as climate change.

While barriers represent opportunities for inclusive interventions, goat enterprises are part of complex and dynamic interconnected livelihood strategies. Holistic strategies are necessary to overcome these barriers in order to support goat enterprise sustainability and development.

## Supplementary Information

Below is the link to the electronic supplementary material.

Supplementary file1 (PDF 251 KB)

Supplementary file2 (XLSX 383 KB)

## Data Availability

The full survey/questionnaire can be found in Supplement A. Raw data of survey responses (with some redactions for anonymity) can be obtained through Mendeley Data (Gwiriri et al., 2025; 10.17632/55xt57vtwg.1).
