# Supplementary material for: Barriers and opportunities to smallholder goat enterprise in Botswana
Source: Food Secur. 2026 Mar 5;18(3):771–90. doi: 10.1007/s12571-026-01648-7 (PMC13183765; doi:10.1007/s12571-026-01648-7)
Supplement: Supplementary file 1 — Supplementary file1 (PDF 251 KB) [file 12571_2026_1648_MOESM1_ESM.pdf]

Supplement A

Below are 8 pages. Of those pages 1 and 2 are the ‘cross-sectional’ survey, whilst pages 3-8 are the ‘semi-structured’ survey.

HOUSEHOLD CHARACTERISTICS — SCREENING QUESTIONNAIRE

**Date & Time**		**GPS (S)**	
**Mobile number**		**GPS (E))**	

Demographic information

	**Respondent**	**Household head**
**Family name**		
**Given name**		
**Sex**	M / F	M / F
**Age group**	<30 / 30-39 / 40-49 / 50-59 / 60<	<30 / 30-39 / 40-49 / 50-59 / 60<
**Education**	None / Form 4-5 (senior) / Primary / Vocational / JC / Tertiary	None / Form 4-5 (senior) / Primary / Vocational / JC / Tertiary
**Literacy**	Yes / No	Yes / No

**Number of family members living here throughout the year**	
**…… of which those regularly engaging in agricultural production**	
**Number of family members living here seasonally or living elsewhere**	

Relative overall importance of the following cash income sources (rank 1, 2, 3…)

**Agriculture**	**Off-farm wage**	**Business**	**Remittance**	**Pension / benefit**

Total number of livestock and their relative overall importance (rank 1, 2, 3…) to the household

	**Goat**	**Sheep**	**Cattle**	**Donkey**	**Horse**	**Chicken**	**Others**
**Number**							
**Rank**							

**In the past 7 days, did you worry that your household would not have enough food?**	Yes / No
**How many meals are taken per day by adults in your households?**	meals
**How many meals are taken per day by children in your households?**	meals

Which of the following is true? Your current income:

Allows you to build savings / Only just meet expenses / Is insufficient so you need to use savings to meet expenses / Is insufficient so you need to borrow to meet expenses

Capital goods ownerships (tick all)

**Donkey cart**	**Bicycle**	**Motor-bike**	**Wheel-barrow**	**Sewing machine**	**Fridge**	**Radio**	**TV**	**Mobile phone**

Number of **goats** by class

Adult female	Adult wether	Adult buck	Kid (< 1 year)

Primary breed

Breed

Who owns the goats?

Household head / Family member living here / Family member living elsewhere / Unrelated employer

How are the goats managed in each season? (tick one)

	Dry season		Rainy season	
	Adults	Kids	Adults	Kids
Free grazing during day, kept in kraal/boma at night				
Free grazing day and night				
Mostly kept in kraal/boma				

Besides grazing, the goats are also fed (tick all):

Grazing only	Grain/meal	Dry forage	Fresh forage	Crop residue	Other
					( )

During the past 12 months, how many goats were added to your household through:

	Birth	Purchase	Gift/government
Adults			
Kids			

During the past 12 months, how many goats were removed from your household through:

	Sales	Home consumption	Gift	Death
Adults				
Kids				

Why do you keep goats? Rank the following reasons by importance (rank 1, 2, 3…)

Sales (cash)	Meat	Milk	Insurance	Other ( )

Do you want to increase your flock size? If so, what is limiting the current flock size? (rank 1, 2, 3…)

Want to?	Cash to buy	Feed	Water	Labour	Other ( )
Yes / No					

What do you use to control parasites in goats? (tick all)

	Ticks	Internal parasites (worms)
Anthelmintic drugs (drench/injection)		
Chemical dip/spray		
Medicinal plants		
Other ( )		

GENERAL DETAILS – INDIVIDUAL QUESTIONNAIRE

1. Do you rotate (change) pastures through the year? YES / NO Why?

2. From what time to what time do your goats spend grazing?

Season	From	To	Total Hours	Days spent grazing per week
Dry				
Wet				

3. Do they come back for water and/or lactation in the middle of the day? YES / NO

   For how long?

4. If your goats are given supplementary feed, please give more details:

Name of feed (from qu. 6)	When given	How much per goat per day	Where from	
			Self-harvested	Bought

5. Are certain plants good to give to sick goats?
   If yes, please state which, for what health problem, and how given.

6. Please rank these characteristics in terms of importance to you in your goats (1 = most important, 5 = least important):

	Fertility	Weight gain	Resistance to illness	Resilience to bad weather	Behaviour / ease of herding
Importance (1 = high / 5 = low)					
Other					

7. How important is goat farming for your and your family's food security?

1 = not important	2 = important	3 = very important	4 =extremely important

8. How often do you drink goat milk (circle)?

   4=Every day / 3 =Every week / 2= Less than once per week / 1 = Rarely or never

9. How important is goat farming as a source of cash income

1 = not important	2 = important	3 = very important	4 =extremely important

10. What percent of your cash income comes from sales of goat products (meat or milk)?

11. Compared to an average household within the village, **your goats** are___ (circle 1-5)

1=less healthy **2**=somewhat less healthy **3**=similar **4**=somewhat more healthy **5**=much more healthy

This is because:

12. Compared to an average household within the village, **your goats** are___ (circle 1-5)

   1=less productive **2**=somewhat less productive **3**=similar **4**=somewhat more productive **5**=much more productive

This is because:

13. According to you, what traits make a productive goat?

14. What currently limits the productivity of your goats?

15. Is sickness/lameness (excluding death) a problem? If so, more problem for adults or kids

16. What is the main health problem for your goats?

17. What kind of health problems occurred in your goats in the past 3 years (tick)?

**Problem:**	**Wet season**	**Dry season**
**Predation**		
**Anaemia**		
**Body condition loss**		
**Diarrhoea**		
**Respiratory disease**		
**Parasites - ticks**		
**Parasites – worms**		
**Heartwater**		
**Other (specify)**		

18. In the past year, how many of your goats died or become sick?

	**Died**		**Sick**	
	Dry season	Rainy season	Dry season	Rainy season
**Young (suckling kids)**				
**Young weaned goats**				
**Adult males**				
**Adult females**				

19. Do you know the effect of ticks on goats' health? YES / NO

If yes, what is it?

20. Do you know the effect of internal parasites (worms) on goats' health? YES / NO

If yes, what is it?

21. If you use anthelmintic drugs, which ones and how do you obtain them?

Anthelmintic drug name	Where obtained	Who pays? (S=self, G=government, O=other)	Application (oral or injection)	How many times per year?	Weigh individual animals to calculate dose? (Y/N)

22. If you apply anthelmintic drugs, which goats receive the treatment (circle)?

All goats Y / N Only goats that look sick? Y / N

23. Do you take faecal samples to estimate level of parasite infection in your goats?

Y / N

If yes, how often?

1=Weekly	2=Monthly	3=Quarterly	4=Yearly

Who does the analysis?

24. How far is the nearest location where anti-parasitic medication can be purchased?

25. Is it affordable to purchase anti-parasitic medication regularly? Y / N

26. Can one afford an once-off (emergency) purchase of anti-parasitic medication? Y / N

27. For farms currently not using anti-parasitic medication:

Why are you not using anti-parasitic medication (tick all that apply)?

1=Cost	2=Lack of availability	3=Other (specify)

28. Do you know medicinal plants used to reduce internal parasites in goats? YES/NO

If yes, please give more details.

29. Do you use medicinal plants for your goats? YES/NO

If yes, how do you feed them?

Which ones, when and for what reason?

Which plants	When (Dry or Wet season)	What ailment

30. Do you know plants that have **negative** impacts in goats? YES / NO

Which plants?

What are the symptoms?

INDIVIDUAL FARMER QUESTIONNAIRE

Is this a project trial farm? Y /N/ Maybe

Questionnaire number:		Interviewer:		Date:	

Management and feeding of goats in Botswana and Malawi

Participant consent form

Please tick the box

1. I confirm that I have read (or someone has read) the questionnaire to me and have understood the summary information contained in the questionnaire. I have had the opportunity to consider the information, ask questions and have had these answered satisfactorily. ☐
2. I understand that my participation is voluntary and that I am free to stop taking part or can withdraw from the study at any time with or without giving any reason and without my rights being affected. In addition, I understand that I am free to decline to answer any particular question or questions. ☐
3. I understand that the information I provide will be held securely and in line with data protection requirements at Botswana International University of Science and Technology (BIUST) where it will be fully anonymised and then deposited in BIUST Research Data Management facility/equivalent only accessible by authorised researchers to support other research in the future. ☐
4. I understand that other authorised researchers may use my words in publications, reports, webpages, and other research outputs, only if they agree to preserve the confidentiality of the information as requested in this form. ☐
5. I understand that I can ask for access to the information I provide and I can request the destruction of that information if I so wish at any time prior to 31/12/19. I understand that following 31/12/19 I will no longer be able to request access to or withdrawal of the information I provide as the data may be published and in the public domain. ☐
6. I understand that signed consent forms and original questionnaires will be retained in BIUST and be digitised for future reference. Original copies will then be destroyed and disposed of securely. ☐
7. I understand that personal information collected about me that can identify me, such as my name, where I live, will not be shared beyond the study team. Confidentiality and anonymity will be maintained and it will not be possible to identify me in any publications. ☐
8. I agree to being contacted at a later date and invited to take part in future studies. I understand that I am only agreeing to receive information and I am under no obligation to take part in any future studies. If you decide not to consent to being contacted in the future it will not have any influence on your involvement in this particular research study. ☐
9. I agree to take part in the above study. ☐

Further note to the person taking consent (if the respondent is unable to provide written consent): I have accurately read out the information sheet to the potential participant and, to the best of my ability, ensured that the participant understands to what they are freely consenting:

___________________________	___ / ____ /____	______________________
**Participant name**	**Date**	**Signature**
___________________________	___ /____ /____	______________________
**Name of enumerator**	**Date**	**Signature**
